# Towards Improving Point-of-Care Diagnosis of Non-malaria Febrile Illness: A Metabolomics Approach

**DOI:** 10.1371/journal.pntd.0004480

**Published:** 2016-03-04

**Authors:** Saskia Decuypere, Jessica Maltha, Stijn Deborggraeve, Nicholas J. W. Rattray, Guiraud Issa, Kaboré Bérenger, Palpouguini Lompo, Marc C. Tahita, Thusitha Ruspasinghe, Malcolm McConville, Royston Goodacre, Halidou Tinto, Jan Jacobs, Jonathan R. Carapetis

**Affiliations:** 1 Telethon Kids Institute, University of Western Australia, Perth, Australia; 2 Department of Clinical Sciences, Institute of Tropical Medicine, Antwerp, Belgium; 3 Center for Molecular and Vascular Biology, KU Leuven, Leuven, Belgium; 4 Department of Biomedical Sciences, Institute of Tropical Medicine, Antwerp, Belgium; 5 School of Chemistry, Manchester Institute of Biotechnology, University of Manchester, Manchester, United Kingdom; 6 Clinical Research Unit Nanoro—IRSS-CRUN, Nanoro, Burkina Faso; 7 Metabolomics Australia and Bio21 Institute of Molecular Sciences and Biotechnology, University of Melbourne, Melbourne, Australia; 8 Department of Immunology and Microbiology, KU Leuven, Leuven, Belgium; University of California San Diego School of Medicine, UNITED STATES

## Abstract

**Introduction:**

Non-malaria febrile illnesses such as bacterial bloodstream infections (BSI) are a leading cause of disease and mortality in the tropics. However, there are no reliable, simple diagnostic tests for identifying BSI or other severe non-malaria febrile illnesses. We hypothesized that different infectious agents responsible for severe febrile illness would impact on the host metabololome in different ways, and investigated the potential of plasma metabolites for diagnosis of non-malaria febrile illness.

**Methodology:**

We conducted a comprehensive mass-spectrometry based metabolomics analysis of the plasma of 61 children with severe febrile illness from a malaria-endemic rural African setting. Metabolite features characteristic for non-malaria febrile illness, BSI, severe anemia and poor clinical outcome were identified by receiver operating curve analysis.

**Principal Findings:**

The plasma metabolome profile of malaria and non-malaria patients revealed fundamental differences in host response, including a differential activation of the hypothalamic-pituitary-adrenal axis. A simple corticosteroid signature was a good classifier of severe malaria and non-malaria febrile patients (AUC 0.82, 95% CI: 0.70–0.93). Patients with BSI were characterized by upregulated plasma bile metabolites; a signature of two bile metabolites was estimated to have a sensitivity of 98.1% (95% CI: 80.2–100) and a specificity of 82.9% (95% CI: 54.7–99.9) to detect BSI in children younger than 5 years. This BSI signature demonstrates that host metabolites can have a superior diagnostic sensitivity compared to pathogen-detecting tests to identify infections characterized by low pathogen load such as BSI.

**Conclusions:**

This study demonstrates the potential use of plasma metabolites to identify causality in children with severe febrile illness in malaria-endemic settings.

## Author Summary

In the tropics, malaria is commonly attributed to be the cause of most childhood fevers, while in fact this condition is more commonly caused by other pathogens that are clinically indistinguishable from malaria. These so-called non-malaria febrile illnesses include bacterial bloodstream infections, which are associated with a higher mortality than malaria. Most health care facilities in the tropics have malaria diagnostic tests available, but tests for non-malarial febrile illnesses are extremely limited. There is the critical need for new tests that can address the question ‘if a febrile patient is not suffering from malaria, then what is it and what treatment will be effective?’ Using metabolomics, we have comprehensively screened the biochemical profile of patients with severe febrile illness for biological markers of non-malaria febrile illness. The results show that severe malaria and non-malaria febrile illness trigger a distinct metabolic response in the host. We demonstrate that this pathophysiological difference can be exploited for differential diagnosis of severe febrile illness and identification of patients with bacterial bloodstream infections.

## Introduction

The introduction of malaria rapid diagnostic tests (RDT) has revealed that febrile illnesses in the tropics and subtropics are more commonly caused by non-malaria pathogens than by malaria [1–5]. Bacterial bloodstream infections (BSI) are considered the most severe non-malaria febrile illness, with mortality rates of 10–25% [6–8]. The high BSI mortality rates highlight the importance of accurate diagnosis and immediate correct case management, particularly in malaria-endemic regions where clinical presentation of BSI and severe malaria are similar [9].

However, in most malaria-endemic regions, children with non-malaria severe febrile illness are not easily diagnosed, which reflects a number of deficiencies in current WHO guidelines that still rely largely on malaria diagnostic tests. First of all, health care workers do not have access to tests to diagnose non-malaria febrile illness, which would inform the most effective treatment. For instance, BSI diagnosis still relies on traditional microbiology, which requires laboratory infrastructure and highly trained staff. Even when available, it takes two to three days for a result, which is too slow to timely inform case management. This can lead to ineffective first-line treatment choices that result in poorer survival outcomes [10], or alternatively leads to over-treatment with broad-spectrum antibiotic therapy that wastes limited resources and fuels emerging antibiotic resistance [11]. Secondly, the current guidelines also fail to alert for primary non-malaria febrile illness in patients with asymptomatic or recent malaria that can have a positive malaria RDT [12]. Finally, the current guidelines fail to recognize concomitant non-malaria febrile illness in patients with severe malaria. BSI/malaria co-infections affect 5–7% of all children with malaria in Africa, and have a 2–3 fold higher mortality rate compared to patients with malaria alone [6].

An ideal test to assess severe febrile illness in malaria-endemic settings should detect more than a single pathogen. In particular, it should provide information on the type of infecting pathogen (malaria and non-malaria) in order to immediately inform on the best treatment and referral options. Such information is unlikely to be captured by a single biological measurement or molecule (biomarker), but is expected to require a combination of molecules (signature). With the development of ‘omics’ profiling approaches it is now possible to measure hundreds of biological analytes simultaneously and thus enable assessment of the diagnostic performance of a molecular signature. This study utilized metabolomics, which in contrast to studies of DNA, RNA and proteins, enables characterization of the metabolome that is the final product of all cell regulatory processes. Hence, a metabolome profile provides a read-out of the (patho)physiological status of a patient at the time of sampling that cannot be obtained directly from the genome, transcriptome, or proteome. In addition, metabolomics analyses only require small sample volumes (20–50 μL) to determine a comprehensive metabolome profile, which is a particular advantage when studying pediatric populations. We hypothesized that the pathophysiological processes triggered by malaria and non-malaria febrile illness induce distinctive changes in the > 4,000 blood metabolites and explored for the first time whether such characteristic metabolites can be used for differential diagnosis of severe febrile illness.

## Materials and Methods

### Ethics statement

The study was conducted according to the principles expressed in the Declaration of Helsinki and was approved by the national ethics committee of Burkina Faso, the institutional review board of the Institute of Tropical Medicine Antwerp, the ethics committee of the University Hospital of Antwerp and the human research ethics office of the University of Western Australia. Written informed consent was given by all parents or guardians of enrolled children.

### Study site, patients and sample collection

Patients were recruited at the district hospital Centre Médical avec Antenne Chirurgicale Saint Camille in malaria-endemic Nanoro, Burkina Faso. Admitted children < 15 years of age presenting with axillary temperature ≥ 38°C, or clinical signs of severe illness were enrolled. Medical history, physical examination and outcome of febrile episode were registered on a standardized form. At time of hospital admission, venous whole blood for blood culture, malaria diagnosis, full blood count, glucose measurements, plasma metabolome analysis, and 16S rRNA deep sequencing were collected from all participants by trained study nurses. Details of sample processing and diagnostic procedures are provided in S1 Methods.

### Case definitions

Malaria was defined as the presence of asexual *Plasmodium falciparum* parasites in blood smear confirmed by microscopy. All patients with a negative blood smear were classified as non-malaria. Recent malaria was defined as positive malaria HRP-2 RDT (which can remain positive up to 6 weeks after successful treatment), but a negative blood smear [13,14]. Confirmed BSI was defined as the growth of clinical significant organisms from blood culture and/or reproducible detection of clinical significant organisms in two 16S deep sequencing experiments. Patients for which 16S deep sequencing could not be performed (n = 5) were classified as ‘BSI diagnosis incomplete’; and patients with unusual but possible clinically relevant bacteria (n = 4) were classified as ‘possible BSI’. Severe anemia was defined as patients with hemoglobin levels < 5 g/dL. Patients who died in hospital following admission for severe febrile illness were classified as non-survival.

### Metabolomics analysis

Mass spectrometry-based metabolomics data were collected for all patients with 3 complementary analytical platforms: gas chromatography mass spectrometry (GC-MS), C_8_ column liquid chromatography mass spectrometry (C_8_-LC-MS) in positive ionization mode, and C_18_ column ultra-high pressure liquid chromatography mass spectrometry (C_18_-UHPLC-MS) in positive and negative ionization mode. These three analytical techniques each capture a different part of the plasma metabolome due to the different chromatography column chemistries used, and thus provide a very broad coverage of the metabolome. Detailed procedures for sample preparation and data acquisition are provided in S1 Methods. Raw data were processed with dedicated data processing pipelines, which are described in S1 Methods. All metabolite data has been deposited in the metabolomics data repository, MetaboLights (study identifier MTBLS315).

### Statistical analysis

All data processing and statistical analyses were performed using the R software environment (version 3.1). This language comprises a selection of packages suitable for the implemented statistical methods: multivariate analysis (partial-least-squares (PLS) regression), receiver-operating curve (ROC) analysis, correlation analysis, hierarchical cluster analysis, biomarker validation and Bayesian latent class modeling. The specific statistical methods and R packages used are explained in S1 Methods, the R scripts can be obtained upon request from the authors.

## Results

We studied the plasma metabolome profile in 61 children with severe febrile illness. All were tested for BSI and malaria. The final diagnosis and characteristics of the patients are shown in Table 1. More than half of the cases in the non-malaria group were diagnosed with community-acquired BSI. The BSI organisms included both Gram-negative and Gram-positive species (Table 2). Non-malaria patients with negative diagnosis for BSI and malaria could be BSI patients that were missed by the used BSI diagnostics that are known to have a limited sensitivity, or could be patients infected with other parasites or viruses that were not standardly tested in the study setting.

**Table 1:** Overview characteristics study participants.

	malaria febrile illness	non-malaria febrile illness		malaria co-infection
	malaria	BSI	negative for BSI & malaria	positive for BSI & malaria
**no. of patients**	30	14	13	4
**% female**	50%	50%	30.8%	50%
mean (range) **age** (months)	39.5 (6–147)	39.5 (0–170)	41 (2–145)	26.6 (2–60)
mean (range) **duration illness** prior to admission (dys)	4.0 (1–7)	4.3 (1–15)	4.4 (1–6)	5 (3–30)
% reported prior **treatment** ^*^	33.3%	64.3%	61.5%	0%
mean (range) **parasite density** (x10^3^ par./mL)	20.8 (0.1–204.2)	0	0	15.1 (2.6–109.4)
mean (range) **Hb** (g/dL)	7.5 (2.2–12.8)	7.5 (3.8–13.5)	7.5 (3.2–15.9)	6.7 (3.8–8.5)
mean (range) **differential neutrophils** (% of total WBC)	51.7 (15.0–90.4)	50.9 (15.5–88.4)	49.1 (19.4–79.6)	43.0 (10.0–55.2)
**% severe malnutrition** ^**^	10%	7%	0	75%
**% non-survival**	10%	50%	38.5%	0%

* Reported prior treatment was defined as reported intake of antimalarial and/or antibiotic treatment in the 48 hrs prior to hospital admission.

** Severe malnutrition was defined as weight for height score < 70% according to national guidelines or report of kwashiorkor.

**Table 2:** Non-malarial clinically significant organisms identified in the study participants.

Clinically significant organisms	No. (%)
all pathogenic bacteria	27 (100%)
*Salmonella enterica*	9 (33.3%)
*Escherichia coli*	4 (14.1%)
*Streptococcus pneumoniae*	3 (11.1%)
*Shigella spp*.	3 (11.1%)
*Enterobacter cloacae*	2 (7.4%)
*Neisseria meningitidis*	2 (7.4%)
*Haemophilus influenzae*	1 (3.7%)
*Klebsiella spp*.	1 (3.7%)
*Staphylococcus aureus*	1 (3.7%)
*Pantoea agglomerans*	1 (3.7%

### The plasma metabolome in patients with severe febrile illness

**Fig 1:**
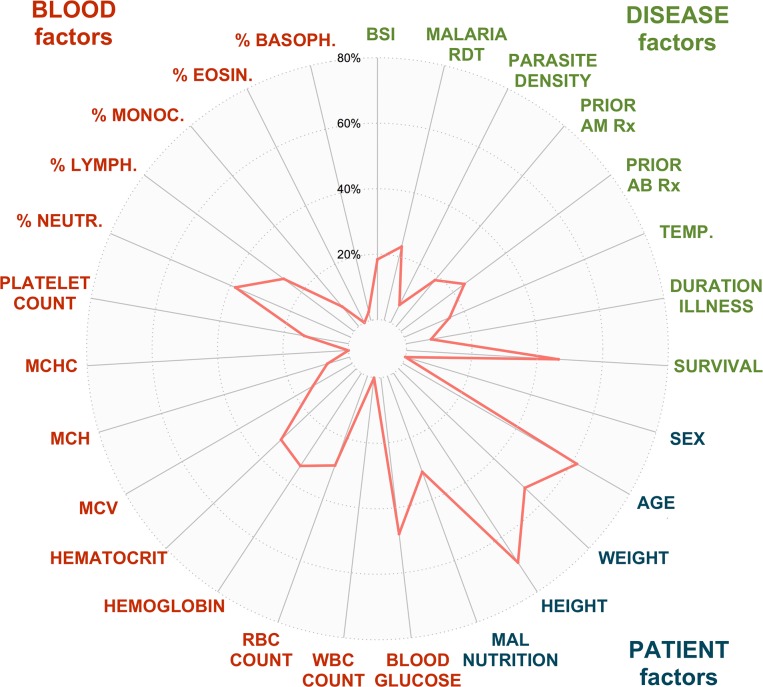
Spiderplot showing the results of the 27 regression analyses modeling the co-variance between metabolome measurements and the listed patient characteristics. The plotted validation metric Q2 allows assessing the relative impact of the patient characteristics on the metabolome. The following 27 patient variables were included in the analysis from center top in clockwise direction: BSI = confirmed or no confirmed BSI diagnosis; malaria RDT = positive or negative malaria rapid diagnostic test; parasite density = number of asexual *Plasmodium* parasites in the bloodstream (x10^3^/mL); prior AB Rx = reported or no reported antibiotic treatment in the past 48 hrs; prior AM Rx = reported or no reported antimalarial treatment in the past 48 hrs; temp. = axillary temperature at time of hospital admission (°C); duration illness = duration illness prior to hospital admission (days); survival = clinical outcome survival or no survival; sex = male or female; age = age expressed in months; weight = weight expressed in kg; height = height expressed in cm; malnutrition = presence or absence of severe malnutrition defined as weight for height score < 70% according to national guidelines or report of kwashiorkor; blood glucose = blood glucose level (mg/dL); WBC = white blood cell count in blood (x10^3^/μl); RBC = red blood cell count in blood (x10^6^/μl); hemoglobin = blood hemoglobin level (g/dL); hematocrit = blood hematocrit (%); MCV = mean corpuscular volume (fL); MCH = mean corpuscular hemoglobin (pg); MCHC = mean corpuscular hemoglobin concentration (g/dl); platelet count = platelet blood count (x10^3^/μL); % neutr. = neutrophils as % of total WBC; % lymph. = lymphocytes as % of total WBC; % monoc. = monocytes as % of total WBC; % eosin. = eosinophils as % of total WBC; % basoph. = basophils as % of total WBC.

A total of 2,635 reproducibly detected plasma *metabolite features* showed a minimal degree of variation (relative standard deviation < 15%) in the study sample (Table 3), these features were further analyzed to fathom the metabolic nature of severe febrile illness. We first investigated with partial-least-squares regression analyses which characteristics of our study participants have a considerable influence on the plasma metabolome composition (Fig 1). Of all the tested patient characteristics, the correlated factors age/weight/height had the biggest impact on the measured metabolome (Q2 = 61.5/53.1/69.3%), closely followed by blood glucose level (Q2 = 48.1%) and disease outcome (survival Q2 = 46.5%). In comparison, the type of infection (BSI, malaria) had little impact on the measured metabolome (Q2 = 18.4% and 23.1% respectively), which does however not preclude the existence of individual metabolite features that are characteristic for the type of infection.

**Table 3:** Overview plasma metabolome coverage.

Mass-spectrometry technology	Coverage plasma of the metabolome	No. of reproducible features with RSD < 15%	No. of features putatively identified	No. of features characterizing severe febrile illness
**GC—MS**	polar metabolites	90	50 (55.6%)	2
**C_8_ LC—MS**	lipids	548	378 (69.0%)	25
**C_18_ UHPLC—MS**	neutral and lipid metabolites	1,997	1,562 (78.2%)	54

Abbreviations: GC-MS = gas chromatography mass spectrometry, C_8_ LC-MS = C_8_ column liquid chromatography mass spectrometry, C_18_ UHPLC-MS = C_18_ column ultra-high pressure liquid chromatography mass spectrometry, RSD = relative standard deviation.

### Metabolic characteristics of severe febrile illness

We identified the metabolite features characterizing the following five patient groups with ROC analysis: (i) patients with non-malaria febrile illness, (ii) malaria patients, (iii) BSI patients, (iv) non-survival patients and (v) severe anemia patients (Table 4). The details of the metabolite features are provided in S1 Data. This section focuses on the metabolite characteristics of non-malaria illness, malaria and BSI; the results for non-survival and severe anemia are described in S1 Results.

**Fig 2:**
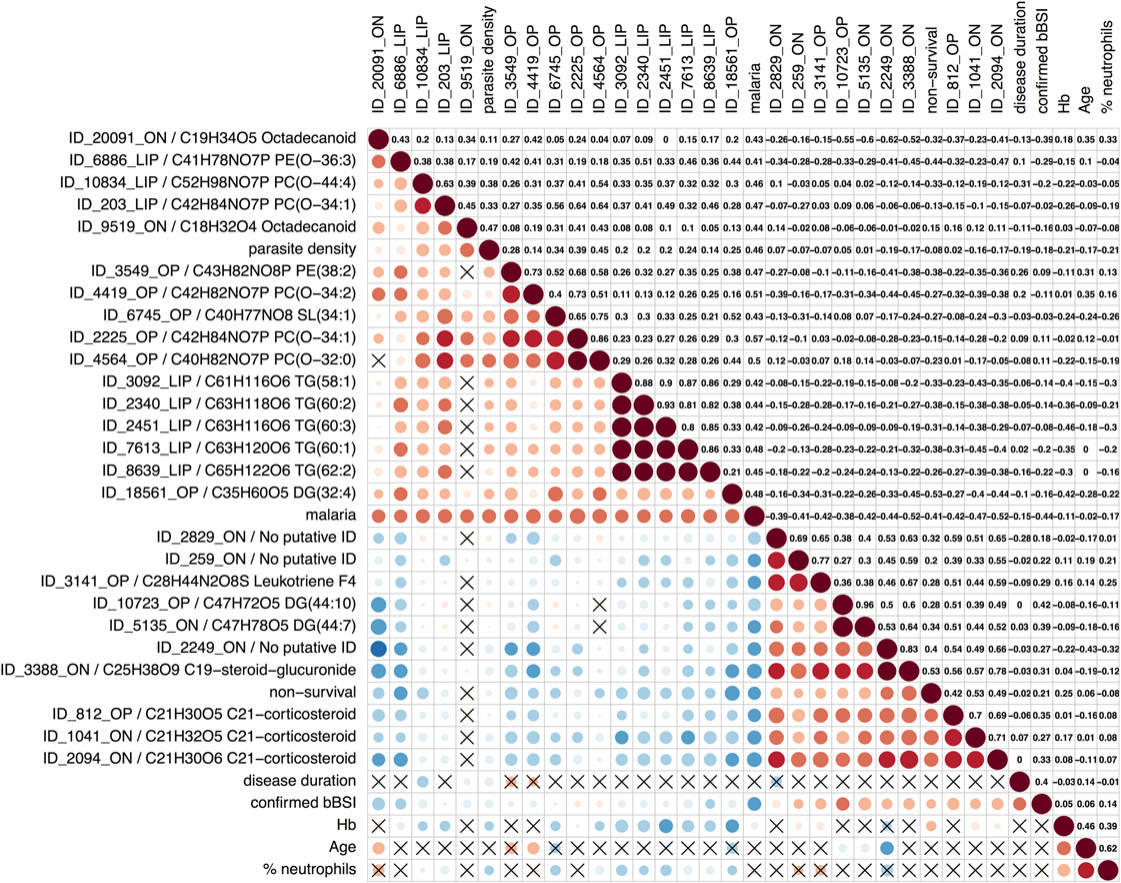
Plot showing correlation between clinical patient data and the metabolite features characterizing non-malaria illness. The plot consists of 2 panels: (i) in the lower panel, the color and size of the circles correspond to the strength of the correlation, with increasing circle size and color intensity indicating increasing correlation; shades of blue are used for negative correlations and shades of red for positive correlations, crosses indicate correlations that were statistically insignificant (p-value < 0.05), and (ii) the upper panel shows the corresponding Pearson’s correlation coefficient r. The left labels for metabolites include the feature ID of the metabolite as listed in S1 data and one putative identification. The following eight patient variables were included: confirmed BSI = confirmed or no confirmed BSI diagnosis; malaria = positive or negative malaria blood smear; parasite density = number of asexual *Plasmodium* parasites in the bloodstream (x10^3^/mL); disease duration = duration illness prior to hospital admission expressed in days; non-survival = clinical outcome survival or no survival; age = age expressed in months; Hb = blood hemoglobin level (g/dL); % neutrophils = neutrophils as % of total WBC. Lipids are abbreviated with PC, PE, SL, DG or TG for phosphatidylcholines, phosphatidylethanolamines, sphingolipids, diacylglycerides and triacylglycerides respectively, and the total number of acyl side-chain carbons and the double bonds in the side-chains.

**Fig 3:**
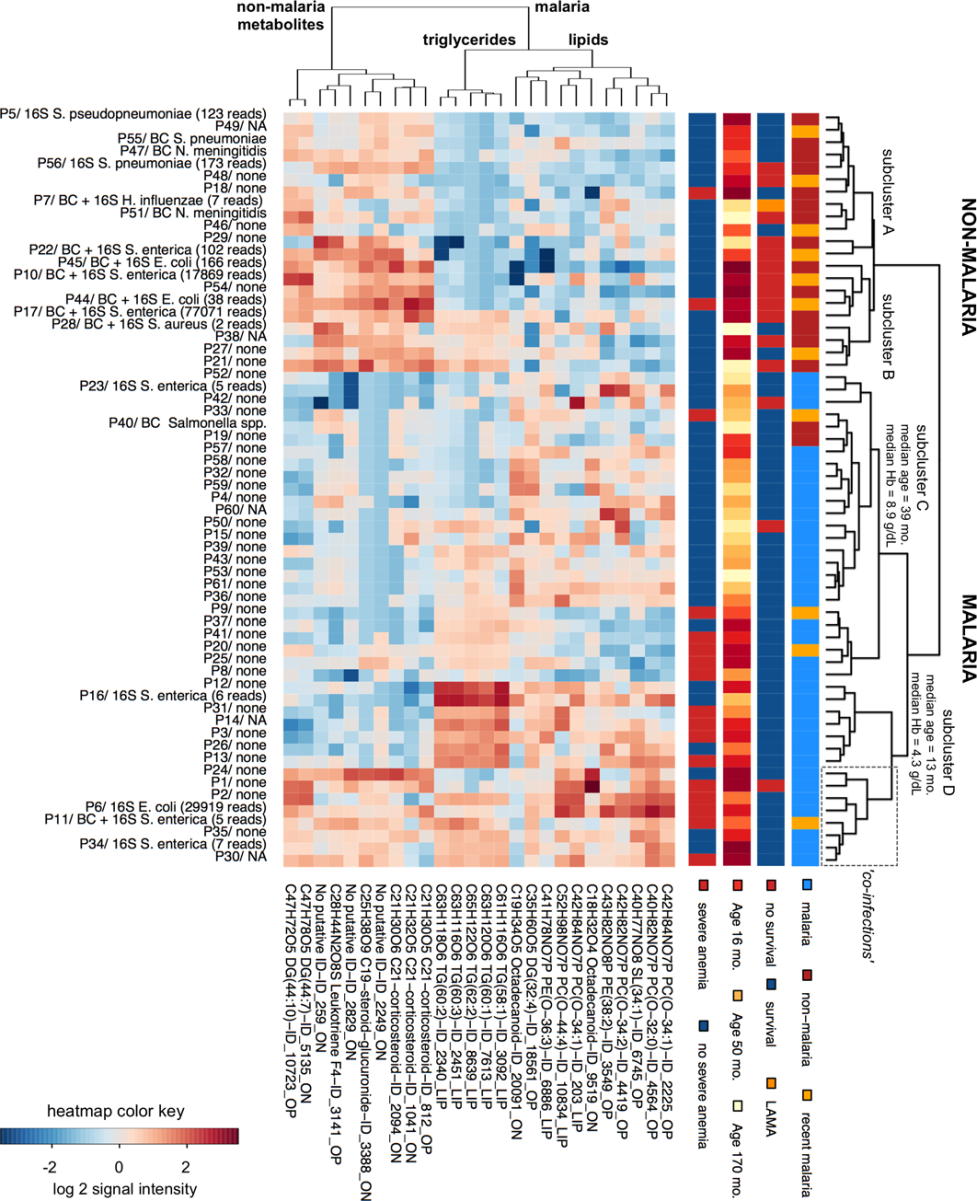
Heatmap of the top 26 features that characterize non-malaria and malaria febrile illness. Patients are shown along the y-axis, the four color-coded bars right of the heatmap indicate their malaria status, survival outcome, age and severe anemia status. The annotation right of the heatmap shows which pathogenic bacteria were identified by blood culture (BC) and/or by 16S sequencing (16S) along with the pathogen species detected (in case of 16S results, only the most abundant pathogen is shown with the matching number of sequencing reads); NA = incomplete BSI diagnosis. The 26 metabolites are shown along the x-axis, their putative identity and feature ID as listed in S1 data are shown below the heatmap. Unsupervised hierarchical clustering of the patients (the tree right of the y-axis) reveals that the shown metabolite intensity profiles differ sufficiently to distinguish non-malaria (upper branch) and malaria patients (lower branch). Clustering of the metabolites according to similarity in intensity profiles (the tree above the x-axis) reveals 3 major groups of metabolite features: the right cluster are plasma phospholipids and fatty acids typically appearing in malaria patients, the middle cluster are the malaria triglycerides, and the left cluster are metabolites characteristic for non-malaria febrile illness. (Abbreviations: LAMA = left against medical advice, mo. = months, Hb = hemoglobin, lipids are abbreviated with PC, PE, SL, DG or TG for phosphatidylcholines, phosphatidylethanolamines, sphingolipids, diacylglycerides and triacylglycerides respectively, and the total number of acyl side-chain carbons and the double bonds in the side-chains.)

We identified a group of 10 correlating metabolite features that characteristically appeared in non-malaria patients (sensitivity range: 0.90–0.67; specificity range: 0.89–0.63; Fig 2). These features include four corticosteroids, of which three were putatively identified as glucocorticoids. The correlation map shows that these corticosteroids were also markers of non-survival patients (S1 Results). In addition, the non-malaria features included three highly correlating features of which one was putatively identified as an eicosanoid (leukotriene F4). Malaria patients on the other hand were characterized by a higher concentration of 16 lipids, predominantly triglycerides and ether phospholipids (sensitivity range: 0.83–0.60; specificity: 0.96–0.63). The latter group included four lipids that showed a fairly positive quantitative correlation with *Plasmodium* parasite density (Pearson’s correlation: 0.38–0.45). The heatmap of the 26 metabolite features characteristic for non-malaria/malaria further clarifies the distinct metabolic character of the two patient groups (Fig 3). Non-malaria patients formed two subclusters. Subcluster A had moderate increases of corticosteroids and predominantly consisted of patients who survived the febrile illness episode, while subcluster B had the highest concentration of corticosteroids seen in our study, and was associated with non-survival. The malaria patients also grouped in two major subclusters. Subcluster C was characterized by more moderate increases of the typical malaria lipids compared to subcluster D which may reflect a differential response that varies with age and/or Hb-levels (higher median age and Hb levels in subcluster C compared to subcluster D). Finally, we expected patients with concomitant malaria and non-malaria febrile illness to have an increase in both the typical malaria lipids/triglycerides and the non-malaria metabolites. Such a cluster of 8 presumptive co-infection patients was indeed observed (far right within subcluster D), of whom two were confirmed as a BSI/malaria co-infection and one as a recent malaria case with a BSI infection.

**Table 4:** Summary metabolite features characteristic for patients with non-malaria, BSI, non-survival and severe anemia.

Phenotype	upregulated features					downregulated features				
	no. features	mean AUC	range fold change	range adjusted p-values	main pathways	no. features	mean AUC	range fold change	range adjusted p-values	main pathways
**non-malaria**27 cases vs 30 controls	10	0.77	1.5–34.2	0.01–0.05	Sterol lipids (corticosteroids)	16	0.77	1.5–12.0	0.01–0.06	Triglycerides & phospholipids
**BSI**18 cases vs 33 controls	8	0.812	1.5–2.9	0.01–0.1	Sterol lipids (bile acids/alcohols)	8	0.79	1.5–3.3	0.07–0.1	Fatty acid oxidation
**non-survival**15 cases vs 45 controls	56 (18)*	0.81	2.4–39.3	0.02–0.05	Sterol lipids (corticosteroids) & amino acids	64 (7)*	0.81	2.01–2.6	0.02–0.05	Triglycerides & phospholipids
**severe anemia**17 cases vs 44 controls	67 (11)**	0.81	1.5–20.7	0.04–0.05	Triglycerides	10 (3)**	0.81	1.6–1.8	0.04–0.05	Fatty acids & sphingolipids

For each analysis, we selected all features with an AUC > 0.75 (excellent to fair classifiers), an adjusted p-value < 0.1 and a fold change in median signal intensity between the cases and controls > 1.5. For the non-malaria analysis, patients with BSI/malaria co-infection were excluded. For the BSI analysis, patients with BSI/malaria co-infection and patients with incomplete or possible BSI diagnosis were excluded. For the non-survival analysis, patients who left hospital against medical advice were excluded. The metabolic features of non-survival and severe anemia patients are further detailed in S1 Results.

* Feature characteristics only shown for the number of features shown between brackets which is the subset of features that have an AUC > 0.75, fold change median signal intensity between 2 compared groups > 2 and p-value < 0.05.

** Feature characteristics only shown for the number of features shown between brackets which is the subset of features that have an AUC > 0.75, fold change median signal intensity between 2 compared groups > 1.5 and p-value < 0.05.

**Fig 4:**
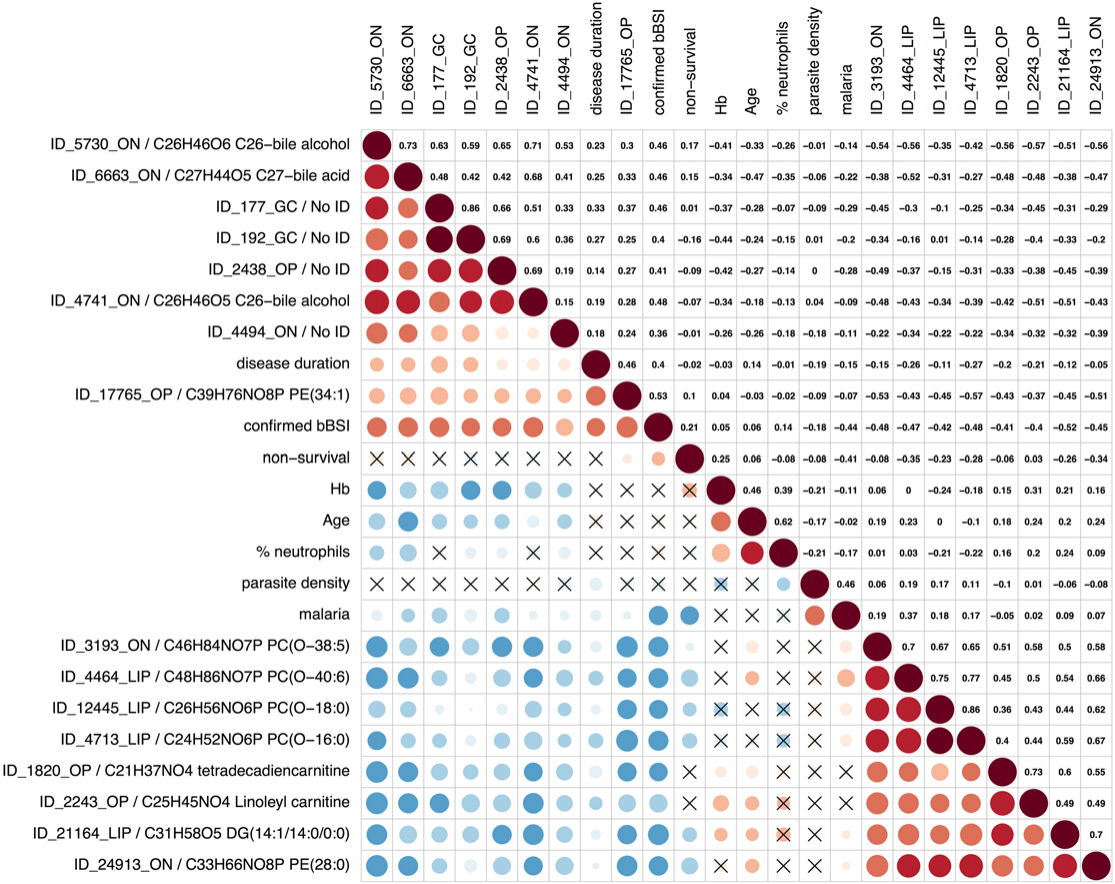
Plot showing correlation between clinical patient data and the metabolite features characterizing BSI. The plot consists of 2 panels: (i) in the lower panel, the color and size of the circles correspond to the strength of the correlation, with increasing circle size and color intensity indicating increasing correlation; shades of blue are used for negative correlations and shades of red for positive correlations, crosses indicate correlations that were statistically insignificant (p-value < 0.05), and (ii) the upper panel shows the corresponding Pearson’s correlation coefficient r. The left labels for metabolites include the feature ID of the metabolite as listed in S1 data and one putative identification. The following eight patient variables were included: confirmed BSI = confirmed or no confirmed BSI diagnosis; malaria = positive or negative malaria blood smear; parasite density = number of asexual *Plasmodium* parasites in the bloodstream (x10^3^/mL); disease duration = duration illness prior to hospital admission expressed in days; non-survival = clinical outcome survival or no survival; age = age expressed in months; Hb = blood hemoglobin level (g/dL); % neutrophils = neutrophils as % of total WBC. Lipids are abbreviated with PC, PE, SL, DG or TG for phosphatidylcholines, phosphatidylethanolamines, sphingolipids, diacylglycerides and triacylglycerides respectively, and the total number of acyl side-chain carbons and the double bonds in the side-chains.

BSI patients were found to have eight upregulated lipids, including several bile acids and alcohols, compared to patients without BSI (sensitivity range: 0.83–0.67; specificity range: 0.91–0.73; Fig 4). We could not find a plausible identification in the checked databases for half of the upregulated features, however given their correlating signal intensity profiles and chromatographic retention-time they were likely biologically and structurally related to the identified bile acids and alcohols. The plasma concentration of these upregulated bile features did not seem to be related to the particular infecting bacterial species.

The metabolome features that had lower concentrations in BSI patients compared to non-BSI patients were consistent with a lower rate of lipolysis (*e*.*g*. lower concentration of lysophospholipids) and a disruption of fatty acid ß-oxidation (*e*.*g*. lower concentration of plasma acylcarnitines).

### Diagnostic signatures for differential diagnosis of severe febrile illness

**Fig 5:**
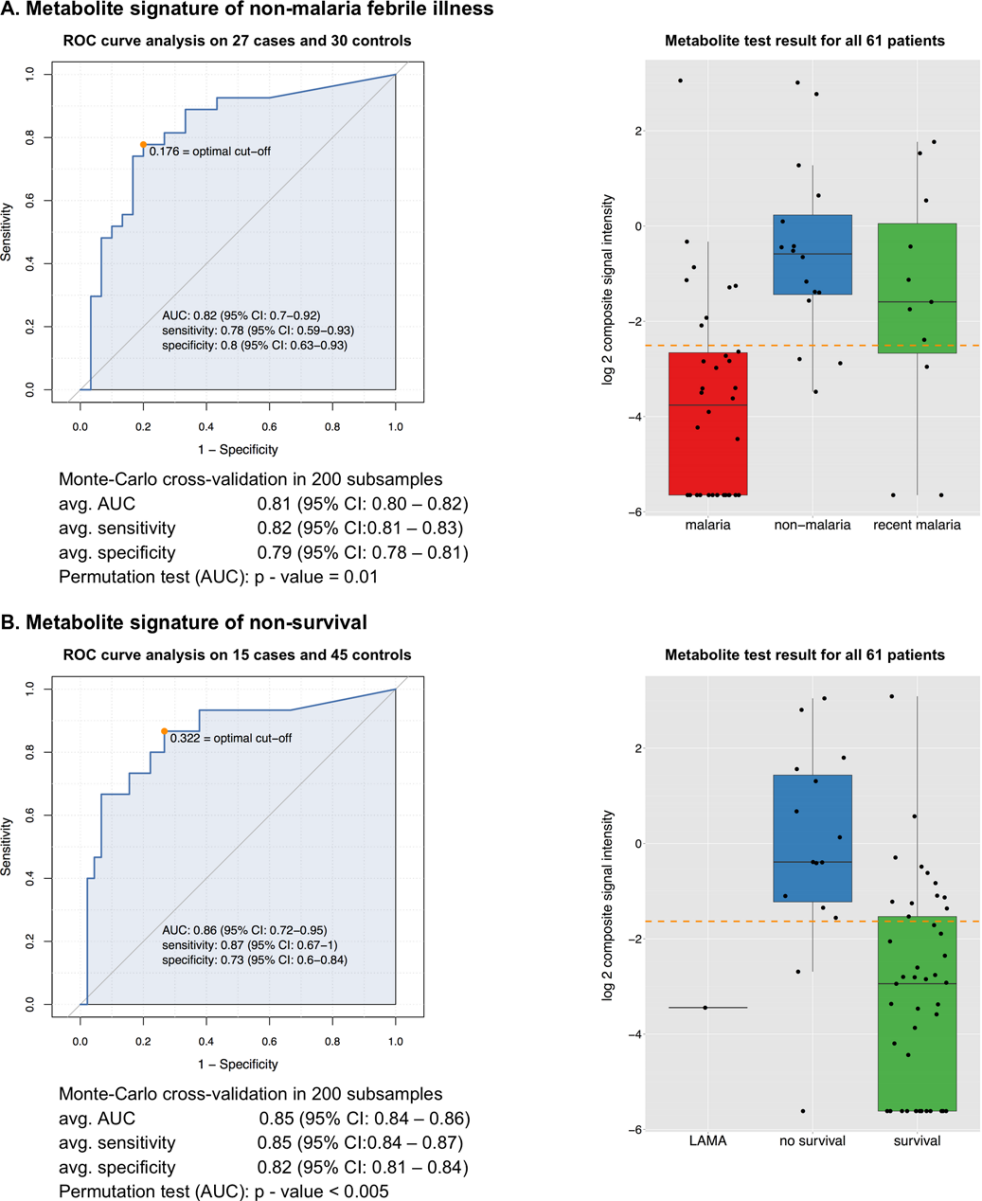
Diagnostic performance of corticosteroid signature for non-malaria febrile illness (panel A) and non-survival (panel B). The metabolite signature consists of 2 corticosteroid compounds detected by C_18_-UHPLC-MS: a C21-corticosteroid (ID_2094_ON, m/z M-H = 377.196, putative ID: C_21_H_30_O_6_ 18-hydroxycortisol) and a C19-steroid-glucuronide (ID_3388_ON, m/z M-H = 481.243, putative ID: C_25_H_38_O_9_ 11-beta-hydroxyandrosterone-3-glucuronide). Each panel shows: (i) a ROC curve along with the optimal cut-off value (the non-malaria analysis excluded BSI/malaria co-infection patients; the non-survival analysis excluded patients that left hospital against medical advice), (ii) check for over-fitting by Monte-Carlo cross-validation based on 200 rounds of balanced subsampling in all cases and controls and the significance permutation test, and (iii) a boxplot with the test result for all 61 patients (x-axis categories indicate the patient groups based on the study case definitions, y-axis shows the test-value of the diagnostic test which is based on the sum of the signal intensity of the two metabolites, here called composite signal intensity, included in each model, the orange dashed line indicates the cut-off value determined in the ROC analysis). (Abbreviations: LAMA = left against medical advice)

**Fig 6:**
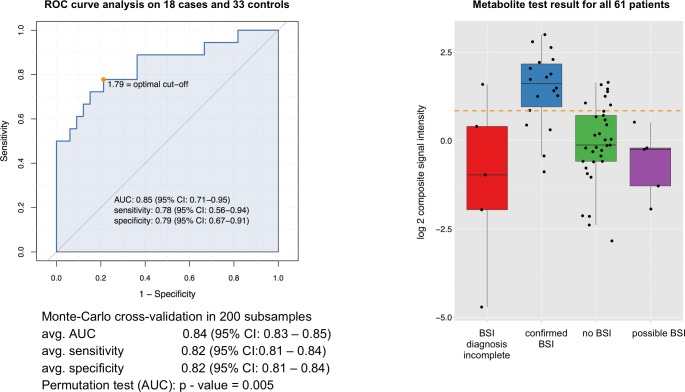
Diagnostic performance of bile metabolite signature for BSI. The metabolite signature consists of 2 bile metabolites detected by C_18_-UHPLC-MS: a C26 bile alcohol (ID_4741_ON, m/z M+FA-H = 483.331, putative ID: C26H46O5 27-Nor-5b-cholestane-3a,7a,12a,24,25-pentol bile alcohol) and a C27 bile acid (ID_6663_ON, m/z M+FA-H = 493.316, putative ID: C27H44O5 C27 bile acid). The figure includes: (i) a ROC curve along with the optimal cut-off value (excluding patients with BSI/malaria co-infection, incomplete or possible BSI diagnosis), (ii) check for over-fitting by Monte-Carlo cross-validation based on 200 rounds of balanced subsampling in all cases and controls and the significance permutation test, and (iii) a boxplot with the test result for all 61 patients (x-axis categories indicate the patient groups based on the study case definitions, y-axis shows the test-value of the diagnostic test which is based on the sum of the signal intensity of the two metabolites, here called composite signal intensity, included in each model, the orange dashed line indicates the cut-off value determined in the ROC analysis).

We selected a signature of two metabolites for non-malaria patients, and also for BSI patients. We assessed the diagnostic performance of the sum of the signal intensity of the 2 individual metabolites for non-malaria febrile illness and BSI respectively (Fig 5 and Fig 6).

The metabolite signature for non-malaria illness consisted of a C21-corticosteroid (m/z M-H = 377.196, putative ID: C_21_H_30_O_6_ 18-hydroxycortisol, ID_2094_ON in S1 Data) and a steroid glucuronide (m/z M-H = 481.243, putative ID: C_25_H_38_O_9_ 11-beta-hydroxyandrosterone-3-glucuronide, ID_3388_ON in S1 Data). The first steroid had an individual area under the curve or AUC = 0.81 (95% CI = 0.68–0.91), adjusted p-value = 0.01, fold change = 4.90; while the second steroid had an AUC = 0.79 (95% CI = 0.67–0.90), adjusted p-value = 0.01 and fold change = 24.6. The ROC curve analysis for the 2 steroids combined including 57 patients (excluding confirmed malaria/BSI co-infections) had a more favorable AUC value of 0.82 (Fig 5A). As shown in the boxplot of Fig 5A, when considering all 61 patients, there were 8 malaria patients falsely classified as non-malaria by this signature, which included six patients that were presumptive malaria/non-malaria co-infections as indicated in subcluster D of Fig 3. The non-malaria signature failed to identify six non-malaria patients, including two BSI patients with grown blood culture.

As explained above, when present at high concentrations the same corticosteroid metabolites were predictive of non-survival. This is confirmed by the ROC-curve analysis on 60 patients (excluding the single patient who left hospital against medical advice) which had a very good AUC of 0.86, and a cut-off value that was indeed almost two-fold higher than the cut-off value to predict non-malaria febrile illness (Fig 5B). A test based on the two combined metabolites missed only 2/15 non-survival patients and thus had a good sensitivity (87%) to predict non-survival. However the specificity (73%) was rather poor, in our patient sample 12/45 survival patients tested false positive (Fig 5B).

We selected a bile acid (m/z M+FA-H = 493.316, putative ID: C_27_H_44_O_5_ C27 bile acid, ID_6663_ON in S1 data) and bile alcohol (m/z M+FA-H = 483.331, putative ID: C_26_H_46_O_5_ 27-Nor-5b-cholestane-3a,7a,12a,24,25-pentol bile alcohol, ID_4741_ON in S1 data) for the BSI signature. The individual diagnostic performance of the bile alcohol was AUC = 0.82 (95% CI = 0.69–0.93), adjusted p-value = 0.07, fold change = 2.54; and for the bile acid AUC = 0.79 (95% CI = 0.63–0.92), adjusted p-value = 0.1, fold change = 2.8. The ROC-curve analysis for the 2 bile metabolites combined including 51 patients (excluding confirmed malaria/BSI co-infections, patients with incomplete or possible BSI diagnosis) had a good AUC of 0.85 (Fig 6). The signature falsely classified 4/18 confirmed BSI cases as non-BSI (Fig 6). Notably these false positives represented 4/5 BSI patients older than 5 years, suggesting that the signature was most suitable to diagnose BSI in under-fives. Eight patients (all malaria patients) in whom we could not detect BSI were classified by the metabolite signature as BSI (Fig 6). It is difficult to conclude whether they were falsely classified by the metabolite signature as BSI-positive, or whether they were misdiagnosed by blood culture/sequencing. We therefore estimated sensitivities and specificities of the three BSI diagnostic tests with Bayesian latent class models (Table 5). Although the confidence intervals were fairly wide given the limited sample size, the results were in line with the expectations. Regardless which age group we considered, the sensitivity of blood culture (50%) was inferior to 16S sequencing (70%). The BSI metabolite signature had the best sensitivity and negative predictive value of the three tests, and reached an impressive 98.1% and 98.3% respectively when considering under-fives only. However, the specificity of the BSI signature (85.8% in all patients, 82.9% in under-fives only) was inferior to that of blood culture and 16S sequencing (> 95% for both tests).

**Table 5:** Estimation of diagnostic performance of BSI tests by Bayesian latent class models.

Diagnostic parameter	BSI tests					
	blood culture *		16S sequencing		BSI signature	
	incl. all patients	incl. <5 yrs only	incl. all patients	incl. <5 yrs only	incl. all patients	incl. <5 yrs only
**Sensitivity (%)**	52.6 (26.6–85.6)	47.3 (23.6–77.9)	71.9 (38.5–99.8)	67.8 (37.9–99.2)	81.4 (54.3–97.7)	98.1 (80.2–100)
**Specificity (%)**	95.7 (80.1–100)	98.2 (84.1–100)	97.6 (81.0–100)	98.6 (84.7–100)	85.8 (62.8–99.9)	82.9 (54.7–99.9)
**PPV (%)**	88.8 (46.9–99.9)	95.3 (58.2–100)	95.3 (54.6–100)	97.4 (68.6–100)	78.2 (37.0–99.9)	80.8 (42.2–99.9)
**NPV (%)**	76.3 (48.6–95.1)	71.3 (45.4–91.8)	84.5 (52.5–99.9)	80.1 (52.3–99.7)	88.2 (62.7–98.7)	98.3 (81.0–100)

The analysis only included patients that had results of all 3 tests. Two analyses were performed (i) including all 51 patients, and (ii) including a subset of 38 patients < 5 years. Results are shown with 95% CI.

* Blood culture was considered the reference method of the test panel; abbreviations used: PPV = positive predictive value, NPV = negative predictive value.

## Discussion

We report here for the first time that malaria and non-malaria severe febrile illnesses each trigger a distinct metabolic host response affecting plasma lipid profiles and this opens new options for differential diagnosis of severe febrile illness. For BSI, one of the most severe non-malaria febrile illnesses, we identified a simple bile metabolite signature with a superior sensitivity and negative predictive value than the current tests (blood culture and 16S sequencing). If our findings can be validated in large-scale studies, then such a simple metabolite signature could be the basis of a new rapid diagnostic test that could potentially reduce BSI mortality by facilitating early diagnosis and timely hospital referral of BSI patients, and reduce empirical antibiotic usage.

Severe malaria was shown to affect the plasma lipid profile, with triglycerides and phospholipids being most significantly changed (Fig 3). Hypertriglyceridemia was also found in all patients with severe anemia (Table 4, S1 Results). A meta-study on the impact of malaria on plasma lipids supports our findings and demonstrated that malaria is characterized by (i) low serum total cholesterol, HDL and LDL which seems to be unique for malaria, and (ii) high triglycerides which is common to other febrile conditions [15]. The underlying biological mechanism of lipid profile changes during malaria is not fully understood yet [15], but may be related to metabolic changes induced by both the parasite and host immune responses. Our data further suggests that some malaria phospholipid markers are moderately quantitatively correlated with parasite density, and that the overall concentration of the typical malaria lipids increases with disease severity (young age and low Hb-levels, Fig 3). Similar findings have been reported by malaria metabolomics and clinical observational studies [16,17], and suggest that host metabolites characteristic for malaria may be more suitable to assess malaria disease progression than to diagnose malaria patients amongst febrile patients.

In contrast to most malaria patients, non-malaria patients were characterized by the presence of immunoregulatory metabolites including corticosteroids, of which the majority were glucocorticoids, and presumably several metabolites related to the eicosanoids. Glucocorticoids are produced by the adrenal glands in response to activation of the hypothalamic-pituitary-adrenal (HPA) axis by the immune system when sensing challenges like infectious agents [18]. During infection these steroid hormones have immuno-suppressive effects to prevent overshooting of inflammatory and immune responses that would be detrimental [19]. Numerous studies have documented HPA activation and subsequent glucocorticoid release upon bacterial and viral infection [18], but little is known about the HPA axis response during malaria [20–24]. One study also reported an inappropriately low glucocorticoid release in severe malaria [24]. The reason for this apparent HPA dysfunction in severe malaria is unclear, but could reflect low levels of the corticosteroid precursor cholesterol seen in malaria (see above), or a deregulation of the pituitary-adrenal function caused by *P*. *falciparum* parasites. Further research is needed to understand this phenomenon, but our results already suggest that whatever the cause of the HPA dysfunction in malaria, it may be overcome by concomitant infections. Indeed, patients with the presumptive co-infections in our study were characterized by both the typical malaria lipids and the immunoregulatory metabolites found in non-malaria illness (Fig 3).

The immunoregulatory metabolites also appeared to be predictive of poor clinical outcome (non-survival) when present in high concentrations (Fig 3 and Fig 5B). There is indeed increasing evidence that an excessive proinflammatory response and an abnormal activation of the HPA axis are key determinants in progression to organ failure and death in patients with critical illness [25,26]. The high circulating levels of glucocorticoids are a symptom of either impaired glucocorticoid clearance or growing glucocorticoid tissue resistance which opens the door to uncontrolled systemic inflammation associated with high mortality [26].

BSI patients were marked by increased plasma levels of bile acids and alcohols, which appeared to be independent of the infecting bacterial species. Elevated plasma levels of bile acids points to endotoxemia-related cholestasis in the liver [27,28], and has also been reported as a marker of sepsis patients with community-acquired pneumonia [25]. Cholestasis and the associated elevated plasma levels of bile metabolites occurs in many conditions affecting liver function but in the target population of severe febrile illness it appeared to be fairly specific for BSI patients. With an estimated sensitivity and specificity > 80%, the BSI bile signature had (i) a superior sensitivity than the current diagnostic tests based on pathogen detection (sensitivity < 70%), (ii) a superior diagnostic performance than clinical assessment (estimated at sensitivity 83%, specificity 62%)[29], and (iii) a performance that very well approaches the reported minimal requirements that should be met by a BSI rapid diagnostic test to be cost-effective in low-resource settings (sensitivity of 83%, specificity of 94%)[29]. The excellent sensitivity (98.1%) and negative predictive value (98.3%) in under-fives is particularly promising as those characteristics would allow the BSI test to be used as a screening test for patients with severe febrile illness attending primary health care service. The results of the test would allow first line health care workers to confidently withhold antibiotics from under-fives that are negative for the bile signature test, while those with a positive test result could be immediately referred to hospital for further diagnosis and care. Such a metabolite screening test needs to meet the ‘ASSURED’ criteria (affordable, sensitive, specific, user- friendly, rapid and robust, equipment free and delivered) to ensure that it can be used in resource-limited and remote settings [30]. Immunoassays generally meet ASSURED and are now available in two formats. The simple lateral-flow “dipstick” test is currently the most widely used format. One test-strip generally carries one or two antibodies for the detection of one or two biomarkers, and different tests can be combined in one device. A new emerging format is the microfluidic chip that allows measurement of multiple biomarkers. The chip is attached to mobile phones to read and display the test results to the healthcare worker. Prototypes of this digital test format are already being tested in Central Africa [31]. Metabolite detection with immunoassays has already been developed by the food & agriculture industry (*e*.*g*. dipstick tests to detect bacterial toxins, pesticides, veterinary drugs) whereby metabolite-binding antibodies are designed with computer-assisted molecular modelling [32].

The design of this study has several limitations. We acknowledge that the presented results are hypothesis-generating and large-scale validation studies are essential to validate the performance of the candidate diagnostic signatures for BSI and non-malaria illness. In this discovery study, we did not include a diagnostic test panel for viral and non-malaria parasitic infections, which could have helped to better characterize the non-malaria patient group. We did not check the identified candidate diagnostic signatures in an asymptomatic control population and in patients with non-severe febrile illness. These 2 groups should be included in future validation studies. However a recent study conducted in Tanzania reported that over 70% of pediatric febrile patients without severe clinical signs had a viral disease [33], thus minimizing the urgency for diagnostic tests for this target population. Hence, we deem it a priority to validate the performance of the non-malaria and BSI signatures in a larger sample of patients with severe febrile illness.

In conclusion, this study demonstrates that malaria and non-malaria patients with severe febrile illness have some fundamental differences in host response that could be exploited for differential diagnosis of severe febrile illness. In combination with the current malaria RDT, a rapid test assessing the plasma levels of 3–4 metabolites could inform on the likelihood of malaria, non-malaria illness, BSI, and survival and could thus empower primary health care centers in making informed treatment and referral decisions.

## Supporting Information

S1 Data An excel sheet listing all the metabolite features (rows) characteristic for non-malaria febrile illness, malaria, non-survival, and severe anemia. The excel sheet comprises the following information in the columns for each metabolite feature: (1) patient group (characterized by metabolite feature), (2) Feature ID (study ID of metabolite feature), (3) area under ROC curve (AUC), (4) sensitivity, (5) specificity, (6) sensitivity + specificity, (7) p-value Mann-Whitney test comparing cases and controls adjusted for multiple comparisons, (8) fold change (= ratio of median signal intensity cases and median signal intensity controls), (9) median signal intensity cases, (10) median signal intensity controls, (11) mass (determined by mass-spectrometry), (12) RT (chromatographic retention time in sec), (13) CoDa-DW (quality value of signal chromatogram determined by the CoDa algorithm which uses the Durbin-Watson statistic), (14) rsd clin. samp. (relative standard deviation of signal intensity in clinical samples), (15) rsd pbqc samples (relative standard deviation of signal intensity in pooled biological quality control samples), (16) formula (chemical formula of assigned putative identifications), (17) putative ID 1 (putative identification 1), (18) KEGG 1 (KEGG entry of putative identification 1), (19) HMDB 1 (HMDB entry of putative identification 1), (20) putative ID 2 (putative identification 2), (21) KEGG 2 (KEGG entry of putative identification 2), (22) HMDB 2 (HMDB entry of putative identification 2), (23) putative ID 3 (putative identification 3), (24) KEGG 3 (KEGG entry of putative identification 3), (25) HMDB 3 (HMDB entry of putative identification 3), (26) class (biochemical class of metabolite feature), (27) remarks on ID (additional information on identifications of metabolite feature), (28) remarks on signal (additional information on quality signal and related peaks = signals corresponding to derivative compounds), (29) MSI scoring (Metabolomics Standards Initiative score of identification with 1 = identified compound, 2 = putatively annotated compounds, 3 = putatively characterized compound classes, 4 = unknown compounds), (30) RT min (chromatographic retention time in min), (31) platform (platform with which the metabolite feature was detected). Metabolite features for which no plausible identification could be found are shaded in grey. Metabolites with a red Feature ID were upregulated in the considered patient group, metabolite features with a blue Feature ID were downregulated in the considered patient group. The putative identifications for the lipids were annotated with the abbreviations PC, PE, SL, MG, DG or TG for phosphatidylcholines, phosphatidylethanolamines, sphingolipids, monoacylglycerides, diacylglycerides and triacylglycerides respectively, and the total number of acyl side-chain carbons and the double bonds in the side-chains.
(XLS)

S1 Methods Additional information on materials and methods including a detailed description of (i) sample processing, (ii) diagnostic procedures used in the study, (iii) metabolomics sample preparation and data acquisition, (iv) metabolomics data processing, and (v) statistical methods. (DOCX)

S1 Results Additional results describing the metabolite features characteristic for patients with severe anemia and in patients with poor clinical outcome (non-survival). (DOCX)
