# Supplementary material for: Towards Improving Point-of-Care Diagnosis of Non-malaria Febrile Illness: A Metabolomics Approach
Source: PLoS Negl Trop Dis. 2016 Mar 4;10(3):e0004480. doi: 10.1371/journal.pntd.0004480 (PMC4778767; doi:10.1371/journal.pntd.0004480)
Supplement: S1 Methods — (DOCX) [file pntd.0004480.s002.docx]

### S1 Methods: additional information materials and methods

Diagnostic procedures

Samples were submitted within 15 min after collection to the lab facility of the Clinical Research Unit of Nanoro (CRUN), which is located on the compound of the district hospital St. Camille.

One to three mL venous blood was collected into a pediatric blood culture bottle (BD BACTEC Preds Plus TM/F, Becton Dickinson and Company, USA). Blood culture bottles were incubated in a BACTEC 9050 instrument (Becton Dickinson) for a total of 5 days. If flagged for growth they were Gram stained, subcultured on EMB agar and 5% Sheep Blood agar (bioMérieux, France) and incubated at 35-37°C for 24 hours in atmospheric conditions and at 5% CO2 respectively. Isolates were identified to the species level by standard biochemical methods.

Thick and thin blood films were stained with 3% Giemsa solution, and examined for presence of *Plasmodium* species according to standard procedures by at least 2 experienced microscopists. Results were expressed as asexual parasites per microliter using the patient’s white blood cell count [[1](#_ENREF_1)].

The malaria rapid diagnostic test (RDT) recommended by the national malaria control program, SD Bioline Pf (Standard Diagnostics, Korea) detecting *P. falciparum*-specific histidine-rich protein-2 (HRP-2), was performed on EDTA blood samples according to the manufacturer’s instructions.

Decision for additional infectious disease testing (*e.g.* HIV) was left to the discretion of attending health staff; only 1 study participant was identified as HIV-positive.

Full blood counts were assessed using Sysmex XS1000i (Sysmex Corporation, Japan). Full blood was collected on NaF tubes for glucose level measurement on Flexor Junior (Vital Scientific, Netherlands).

For metabolomics analysis, one 1 ml EDTA blood sample was centrifuged at 3,000 g for 20 min at 4°C for plasma separation. The plasma fraction was divided in 100 µL aliquots and immediately frozen at -80°C. Plasma aliquots were stored within 2 hours of sample collection, plasma processing times were kept in the study log book. Plasma samples were shipped on dry ice with temperature-control to the Institute of Tropical Medicine Antwerp for metabolite extraction.

Leftover EDTA blood samples (200 – 1000 µL) were kept at -80°C at CRUN and utilized for additional molecular diagnosis of BSI. PCR products resulting from amplification of the V3-V4 regions of the bacterial 16S rRNA gene were deep sequenced on the Illumnia MiSeq platform (Illumina, USA). The sequencing reads underwent a quality filtering process using mothur [[2](#_ENREF_2)]. Paired reads were joined into single contigs and any contigs with ambiguous bases or a length longer than the 2 individual read lengths, were removed. Taxonomy was assigned using GAST [[3](#_ENREF_3)] and the associated full length 16S SILVA reference database (retrieved 13/02/15). Taxonomy assignments found within any negative control samples were labeled as potential contaminants and removed from all samples. Samples were only considered positive if a taxonomy assignment up to species level of a clinically significant organism could be reproduced in 2 repeated sequencing experiments.

Metabolomics sample preparation and data acquisition

Samples for GC-MS and UHPLC-MS were prepared as described previously [[4](#_ENREF_4)] with minor modifications. Briefly, a 50 µL plasma aliquot was thawed on ice followed by addition of 100 µL ultrapure methanol. For GC-MS, an additional 100 µL internal standard solution (0.2 mg/mL succinic-d_4_ acid, benzoic d_5_ acid, glycine-d_5_, lysine-d_4_ from Cambridge Isotope laboratories, UK) was added. The sample was vortex mixed and centrifuged at 13,500 rpm for 15 min. Following centrifugation, 125 µL of the clean supernatant was transferred to a clean tube and dried in a centrifugal vacuum evaporator for 16 hrs. Samples for lipid analysis on C_8_-LC-MS positive mode were prepared with a butanol/methanol extraction. The extraction solvent was prepared by mixing equal amounts of pure butanol and methanol, 10 mM ammonium formate, 0.1% butylated hydroxytoluene and 2.5 µM internal standard solution including D_31_PE(34:1) , D_31_PS(34:1) and D_31_PI(34:1) (Avanti Polar Lipids Inc., USA ). A 10 µL plasma aliquot was thawed on ice and mixed with 90 µL of the extraction solvent. The mixture was vortexed for 30 sec and sonicated for 30 min, all the while ensuring that temperature was maintained at room temperature (20°C). The resulting mix was centrifuged at 13,000 rpm for 15 minutes at 0°C, and 50 µL of the supernatant was directly transferred to 0.2 mL micro-inserts of LC-MS glass vials with Teflon insert caps.

Quality control samples were prepared together with patient samples to enable monitoring of (i) introduction of contaminants during sample preparation (extraction blanks), and (ii) stability of platform performance during analytical run (aliquoted pooled sample of all patient samples, further referred to as QC samples).

Data acquisition on GC-MS and UHPLC-MS was performed as described previously by Dunn et al. [[4](#_ENREF_4)] with some modifications. Briefly, samples for GC-MS analysis were prepared applying a two-stage chemical derivatisation procedure (oximation followed by trimethylsilylation) and followed by analysis applying an electron ionisation GC-TOF-MS system (Agilent 6890N GC coupled to a LECO Pegasus III mass spectrometer). For GC-MS, 5 QC samples were analysed at the start of each analytical batch to condition the analytical system and a QC sample was analysed every 5th injection. The study samples were analysed in 2 analytical experimental batches. For UHPLC-MS, samples were reconstituted in 50 µL water for positive and negative mode and analysed applying C_18_-UHPLC-Orbitrap MS (Thermo Accela UHPLC coupled to a Thermo LTQ Orbitrap mass spectrometer) over a 25 min runtime. Ten QC samples were analysed at the start of an analytical batch to condition the analytical system and a QC sample was analysed every 5^th^ injection. The study samples were analysed in 1 analytical batch. For C_8_-QTOF-MS, the samples were analysed as described earlier [[5](#_ENREF_5)] on a C_8_-LC-QTOF (Agilent 1290 LC coupled to an Agilent QTOF 6550 mass spectrometer) with a 30 min runtime. Ten solvent samples were analysed at the start of the analytical batch to condition the system and a QC sample was analysed every 4^th^ injection. All study samples were analysed in one analytical batch. Samples were analysed in randomized order in each experiment.

Metabolomics data pre-preprocessing

The two batches of raw GC-ToF-MS data files were peak picked and deconvolved using an internal deconvolution algorithm within the ChromaTof 2.1 software (Leco). This was applied stipulating an increasing peak width of 1.5s (at 400s) expanding to 1.8s (at 800s), a baseline cut-off at 0.5 and aligning to an internal retention index (decane, dodecane, pentadecane and nonadecane). Once deconvolved, metabolite identification was performed initially against an in-house metabolite reference database of over 1500 internal standards (generally linked to primary metabolism) and then subsequently against putative mass spectral fragmentation patterns within the NIST 8.0 spectral database (containing mass spectra from over 800,000 different compounds). In order to account for instrumental drift, an in-house LOESS correction curve was splined through each QC data point and each analytical point aligned accordingly [[4](#_ENREF_4)]. This procedure also aligned both GC-ToF-MS batches.

The LC-MS raw data files were converted into the mzXML format by msConvert, a tool from the ProteoWizard toolkit for converting between various file formats [[6](#_ENREF_6)]. Further processing was handled by a flexible data processing pipeline implemented in R called mzMatch.R [[7](#_ENREF_7)] (http://mzmatch.sourceforge.net/) which includes XCMS-based mass chromatogram extraction and retention time alignment [[8](#_ENREF_8)], peak matching, noise removal [[9](#_ENREF_9)], contaminant removal based on extraction and solvent blanks, implementation dilution trend correlation filter to further remove non-informative peaks [[10](#_ENREF_10)], correction signal drift over time based on fitting a LOESS function on the data of the QC samples [[4](#_ENREF_4)], and peak derivative removal (isotopes, adducts, dimers and fragments) based on correlation analysis on both peak shape and intensity pattern [[11](#_ENREF_11)].

Metabolite peaks were considered to be detected reproducibly when they were detected in all QC samples and when the quantitative variation of the signal intensity across all the QC samples was less than 15% relative standard deviation (RSD) for the LC-MS platforms (samples analyzed in 1 analytical batch) and less than 20% RSD for GC-MS (samples analysed in 2 analytical batches). All peaks that did not fulfill these criteria were removed. Non-informative peaks that would not yield interesting biomarkers were also removed from the dataset and included: (i) peaks with signal intensity close to the limit of detection, (ii) peaks detected in less than 3 out of the 61 study samples, (iii) peaks that are near-constant in all included patient samples (RSD <15%). The final peak set obtained for each LC-MS platform was visually inspected to ensure that all included peaks corresponded to good-quality mass- chromatograms. The selected peaks were putatively identified by matching the masses (mass accuracy < 5 ppm) progressively to those from metabolite-specific databases. In a first round of identification, we used the Human Metabolome Database [[12](#_ENREF_12)], KEGG [[13](#_ENREF_13)], and a contaminant database [[14](#_ENREF_14)]. Only the remaining unidentified peaks went through a second round of matching to LIPIDMAPS [[15](#_ENREF_15)] and METLIN spectral database [[16](#_ENREF_16)]. Missing values in the data-matrix obtained per analytical platform were replaced with half the minimum value of the entire matrix of that analytical batch. The final metabolite features (*i.e.*, peaks that could be identified to one of the four levels designated by the Metabolomics Standards Initiative [[17](#_ENREF_17)]) of all analytical platforms were merged into 1 data-matrix that was checked in R for outlying samples using standard chemometric tools including principal component analysis (R package pcaMethods) and Mahalanobis distance (R package chemometrics). No outliers were detected and all samples were included in subsequent statistical analysis.

Statistical methods

Partial-least squares regression (PLS) analysis was used to capture the variation in the metabolite matrix that is important to predict the variation of a patient characteristic. The result is provided by the parameter *Q^2^* which tells us how good the prediction of Y is based on the cross-validated PLS model (R package pls). We note that *Q*^2^ is a validation metric that is better used for regression analysis [[18](#_ENREF_18)] and as seen in Figure 1 many of our patient characteristics are quantitative in nature. *Q*^2^ in this study is only used to assess the impact of the patient on the metabolome which can be affected by extrinsic and intrinsic factors [[19](#_ENREF_19)] and was not used for classification per se.

Multivariate data modeling methods such as PLS perform better if highly correlated features are removed, hence we identified and removed all highly correlating (Pearson’s correlation > 0.75, R package caret) features before proceeding to PLS.

The area under the receiver-operating curve (ROC) was used to identify metabolite features characteristic for specific patient groups (R packages ROCR and pROC). The results of the ROC curves were summarized with the single metric Area Under Curve (AUC) and the equivalent Mann-Whitney U statistic. AUC indicates the probability that a classifier will rank a randomly chosen case higher than a randomly chosen control. We compensated for multiple comparisons by adjusting the Mann-Whitney p-values with Benjamini-Hochberg corrections. For the analysis of a specific patient group (non-malaria, BSI, non-survival, severe anemia) we assigned the study participants to either cases or controls; patients that could not be classified as either case or control were excluded. We determined the ROC curve for every metabolite feature and selected all features with an AUC > 0.75 (excellent to fair classifiers), an adjusted p-value < 0.1 and a fold change in median signal intensity between the cases and controls > 1.5. We verified the putative identification of the selected features and derived which major metabolic changes characterize each patient group.

The strength and direction of association that exists between 2 continuous variables was assessed with Pearson’s correlation (R package stats), and the results visualized in correlation plots (R package corrplot). Pearson’s correlation between paired variables is expressed in Pearson’s correlation coefficient r: a value between 0.5 - 1 corresponds to high correlation, 0.3 - 0.5 corresponds to medium correlation, and 0.1 - 0.3 corresponds to low correlation.

Unsupervised hierarchical clustering and heatmap plotting was used to identify groups of samples or variables that behave similarly or show similar characteristics (R package stats and gplots). Hierarchical clustering algorithms build an entire tree of nested clusters out of objects in the dataset by an iterative clustering algorithm [[20](#_ENREF_20)].

Monte Carlo cross-validation allows assessing what the robustness of the diagnostic test performance would be in an independent sample set without actually having an independent new sample set. MCCV (or repeated random sub-sampling validation) splits the study sample randomly into balanced subsamples. For each split, the performance of the diagnostic signature under evaluation is evaluated on the subsample by ROC curve analysis. This subsampling and ROC assessment is repeated 200 times. A diagnostic signature that shows a highly reproducible AUC value over all iterations is considered a robust new candidate diagnostic test.

Permutation testing proves or disproves the null hypothesis that the diagnostic signature identified during the biomarker discovery process could also have been found if each patient sample had been randomly assigned a clinical status in the same proportion as in the true assignment. The first step in a permutation test is to randomly permute the clinical status labels of the samples, and then similarly as in MCCV, the diagnostic signature is iteratively evaluated by ROC curve analysis on balanced subsets of the permuted sample. The AUC-values of the permuted samples are considered a null distribution of AUC-values. Statistical significance of the diagnostic signature can then be assessed by comparing the distribution of AUC-values obtained in MCCV to the null distribution obtained by permutation tests. A p value < 0.05 means that given a randomly permuted outcome variable there is less than a 5% chance that a diagnostic signature of similar performance to the true non-permuted model will be produced.

Bayesian latent class models (LCMs) were used to estimate and compare the true diagnostic accuracy of the candidate diagnostic signatures with existing diagnostic tests. LCM is a statistical modeling approach that does not assume a single gold standard test, but regards each conducted test as imperfect in diagnosing the true disease status, which are the 2 latent variables in the model. LCMs are particularly recommended to estimate the diagnostic accuracy of a new test when the gold standard diagnostic test is known to have possible accuracy issues [[21](#_ENREF_21)]. We fitted a 2-class model for the BSI diagnostics and assumed that blood culture and 16S sequencing are correlated (R package WinBugs).

**References S1 Methods:**

1. Organisation WH. Basic Malaria Microscopy - Part I. Learner's Guide., 1991.

2. Kozich JJ, Westcott SL, Baxter NT, Highlander SK, Schloss PD. Development of a dual-index sequencing strategy and curation pipeline for analyzing amplicon sequence data on the MiSeq Illumina sequencing platform. Appl Environ Microbiol 2013; 79(17): 5112-20.

3. Huse SM, Dethlefsen L, Huber JA, Mark Welch D, Relman DA, Sogin ML. Exploring microbial diversity and taxonomy using SSU rRNA hypervariable tag sequencing. PLoS Genet 2008; 4(11): e1000255.

4. Dunn WB, Broadhurst D, Begley P, et al. Procedures for large-scale metabolic profiling of serum and plasma using gas chromatography and liquid chromatography coupled to mass spectrometry. Nat Protoc 2011; 6(7): 1060-83.

5. Hu C, van Dommelen J, van der Heijden R, et al. RPLC-ion-trap-FTMS method for lipid profiling of plasma: method validation and application to p53 mutant mouse model. J Proteome Res 2008; 7(11): 4982-91.

6. Chambers MC, Maclean B, Burke R, et al. A cross-platform toolkit for mass spectrometry and proteomics. Nat Biotechnol 2012; 30(10): 918-20.

7. Scheltema RA, Jankevics A, Jansen RC, Swertz MA, Breitling R. PeakML/mzMatch: a file format, Java library, R library, and tool-chain for mass spectrometry data analysis. Anal Chem 2011; 83(7): 2786-93.

8. Smith CA, Want EJ, O'Maille G, Abagyan R, Siuzdak G. XCMS: processing mass spectrometry data for metabolite profiling using nonlinear peak alignment, matching, and identification. Anal Chem 2006; 78(3): 779-87.

9. Windig W. The use of the Durbin-Watson criterion for noise and background reduction of complex liquid chromatography/mass spectrometry data and a new algorithm to determine sample differences. Chemometrics and Intelligent Laboratory Systems 2005; 77(1–2): 206-14.

10. Jankevics A, Merlo ME, de Vries M, Vonk RJ, Takano E, Breitling R. Separating the wheat from the chaff: a prioritisation pipeline for the analysis of metabolomics datasets. Metabolomics : Official journal of the Metabolomic Society 2012; 8(Suppl 1): 29-36.

11. Scheltema RA, Decuypere S, Dujardin JC, Watson DG, Jansen RC, Breitling R. Simple data-reduction method for high-resolution LC-MS data in metabolomics. Bioanalysis 2009; 1(9): 1551-7.

12. Wishart DS, Jewison T, Guo AC, et al. HMDB 3.0--The Human Metabolome Database in 2013. Nucleic Acids Res 2013; 41(Database issue): D801-7.

13. Ogata H, Goto S, Sato K, Fujibuchi W, Bono H, Kanehisa M. KEGG: Kyoto Encyclopedia of Genes and Genomes. Nucleic Acids Res 1999; 27(1): 29-34.

14. Keller BO, Sui J, Young AB, Whittal RM. Interferences and contaminants encountered in modern mass spectrometry. Anal Chim Acta 2008; 627(1): 71-81.

15. Fahy E, Sud M, Cotter D, Subramaniam S. LIPID MAPS online tools for lipid research. Nucleic Acids Res 2007; 35(Web Server issue): W606-12.

16. Smith CA, O'Maille G, Want EJ, et al. METLIN: a metabolite mass spectral database. Therapeutic drug monitoring 2005; 27(6): 747-51.

17. Sumner LW, Amberg A, Barrett D, et al. Proposed minimum reporting standards for chemical analysis Chemical Analysis Working Group (CAWG) Metabolomics Standards Initiative (MSI). Metabolomics : Official journal of the Metabolomic Society 2007; 3(3): 211-21.

18. Westerhuis JA, Hoefsloot HCJ, Smit S, et al. Assessment of PLSDA cross validation. Metabolomics : Official journal of the Metabolomic Society 2008; 4(1): 81-9.

19. Goodacre R. Metabolomics of a superorganism. The Journal of nutrition 2007; 137(1 Suppl): 259S-66S.

20. Eisen MB, Spellman PT, Brown PO, Botstein D. Cluster analysis and display of genome-wide expression patterns. Proc Natl Acad Sci U S A 1998; 95(25): 14863-8.

21. Menten J, Boelaert M, Lesaffre E. Bayesian latent class models with conditionally dependent diagnostic tests: a case study. Stat Med 2008; 27(22): 4469-88.
