# Supplementary material for: Towards Improving Point-of-Care Diagnosis of Non-malaria Febrile Illness: A Metabolomics Approach
Source: PLoS Negl Trop Dis. 2016 Mar 4;10(3):e0004480. doi: 10.1371/journal.pntd.0004480 (PMC4778767; doi:10.1371/journal.pntd.0004480)
Supplement: S1 Results — (DOCX) [file pntd.0004480.s003.docx]

**S1 Results: Metabolite features of non-survival and severe anemia**

*Metabolite features of non-survival*

When using the set AUC-based selection criteria, we obtained a list of 120 metabolite features characterizing non-survival patients. This large number of characteristic features shows -as already discussed in the PLS analysis (Fig 1)- that the final clinical disease outcome had a far larger impact on the plasma metabolome than the type of infection. We verified the identification and matching metabolic pathways of a subset of 25 features with a p-value < 0.05 and fold change in median signal intensity between the cases and controls > 2 (S1 Data).

Non-survival was characterized by a higher concentration of 2 groups of correlating plasma metabolites (sensitivity range: 0.93 – 0.67, specificity range: 0.91 – 0.64; S1 Figure). The first group consisted mainly of proteolysis products (*e.g.* peptides, products of amino acid catabolism) and some products that presumably resulted from cell membrane disintegration and lipid catabolism (*e.g*. acyl glycines, ganglioside). The second group consisted of corticosteroids and their catabolic products.

Survival was characterized by a cluster of correlating metabolites that mainly consists of di- and triglycerides.

|  |
| --- |
| ***Figure A. Plot showing correlation between clinical patient data and the metabolite features characterizing non-survival patients.*** *The colors and size of the circles correspond to the strength of the correlation, with increasing circle size and color intensity indicating increasing correlation. Shades of red are used for negative correlations and shades of blue for positive correlations. Only one putative identification shown. (Abbreviations: PD = parasite density, DisDur = disease duration, DifNeu = differential neutrophil count, Hb = hemoglobin, lipids are abbreviated with PC and MG for phosphatidylcholines and monoacylglycerides respectively, and the total number of acyl side-chain carbons and the double bonds in the side-chains)* |

*Metabolite features of severe anemia*

For severe anemia, we obtained a total of 78 features when using the set AUC-based selection criteria; this was again in line with the PLS results which showed that hemoglobin level indeed exerted a considerable influence on the plasma metabolome composition (Fig 1). We checked the identification and matching metabolic pathways of a subset of 14 features with p-value < 0.05 (S1 Data).

The upregulated features were all glycerides, predominantly triglycerides with highly correlating profiles (sensitivity range: 0.94 – 0.65, specificity range: 0.91 – 0.61, S2 Figure). The triglyceride levels had a strong negative correlation with Hb-levels, and by association a weaker correlation with age and differential neutrophil count.

Only a few downregulated features were found in our dataset, these were again lipids, but not glycerides.

|  |
| --- |
| ***Figure B. Plot showing correlation between clinical patient data and the metabolite features characterizing severe anemia (hemoglobin levels < 5 g/dL).*** *The colors and size of the circles correspond to the strength of the correlation, with increasing circle size and color intensity indicating increasing correlation. Shades of red are used for negative correlations and shades of blue for positive correlations. Only one putative identification shown. (Abbreviations: PD = parasite density, DisDur = disease duration, DifNeu = differential neutrophil count, Hb = hemoglobin, lipids are abbreviated with MG, DG or TG for monoacylglycerides, diacylglycerides and triacylglycerides respectively, and the total number of acyl side-chain carbons and the double bonds in the side-chains)* |
